# Supplementary material for: Empowering Women, Enhancing Health: The Role of Education in Water, Sanitation, and Hygiene (WaSH) and Child Health Outcomes
Source: Int J Environ Res Public Health. 2025 Apr 29;22(5):706. doi: 10.3390/ijerph22050706 (PMC12111128; doi:10.3390/ijerph22050706)
Supplement: Supplementary file 1 [file ijerph-22-00706-s001.zip › ijerph-3398768-supplementary.pdf]

VIF values of Model II from Table 2

1.114024 1.463403 1.150766 1.117575 1.052242 1.555862

VIF values of Model II from Table 3

1.179513 1.672003 1.274470 1.126793 1.780201 2.242749

VIF values of Model II from Table 4

1.373587 1.235928 1.315312 1.125369 1.123275 1.907230

VIF values of Model IV from Table 2

2.287499 1.723672 1.264799 1.156769 1.108527 1.087297 1.075342 1.056256 1.040402 1.004469

VIF values of Model IV from Table 3

2.461793 1.713738 1.308856 1.223529 1.130778 1.111716 1.072678 1.064488 1.047034 1.042067 1.004481

VIF values of Model IV from Table 4

1.927205 3.632420 1.633974 1.330762 1.194564 1.160070 1.119336 1.109978 1.063418 1.057703 1.005359

Normal Q-Q Plot of Model II from Table 2

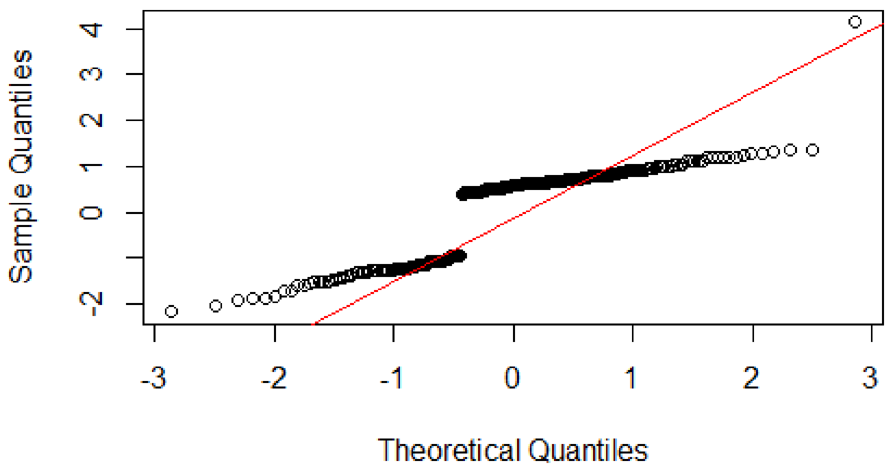

Normal Q-Q Plot of Model II from Table 3

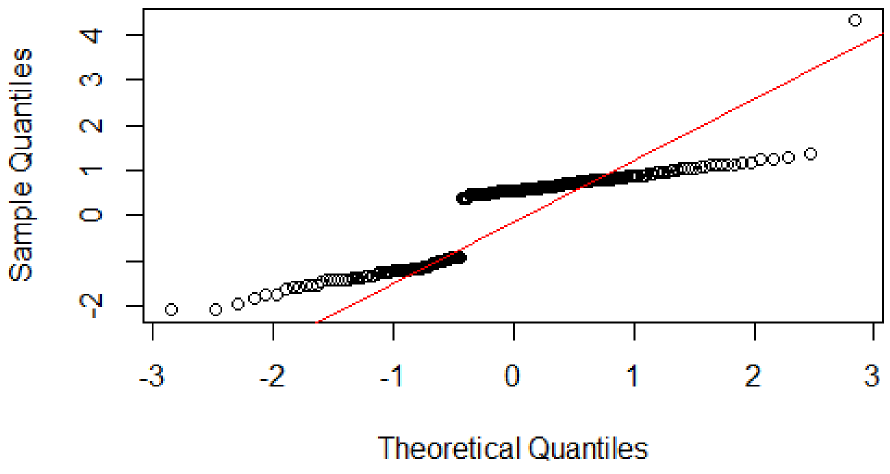

Normal Q-Q Plot of Model II from Table 4

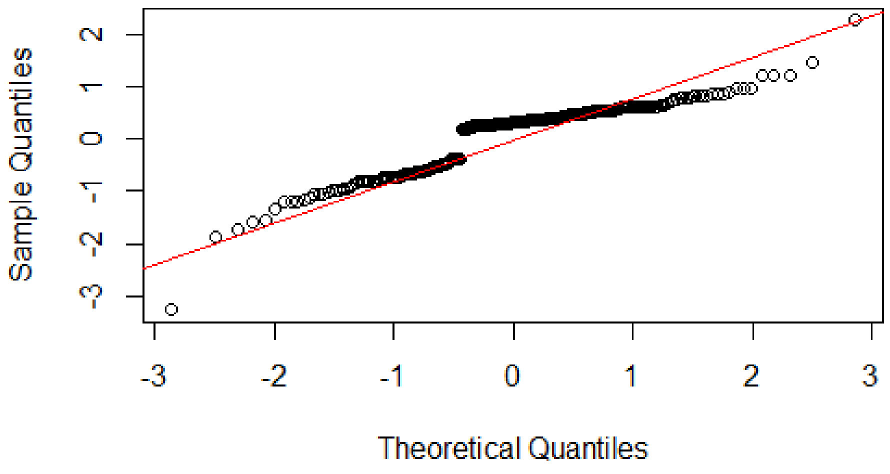

Normal Q-Q Plot of Model IV from Table 2

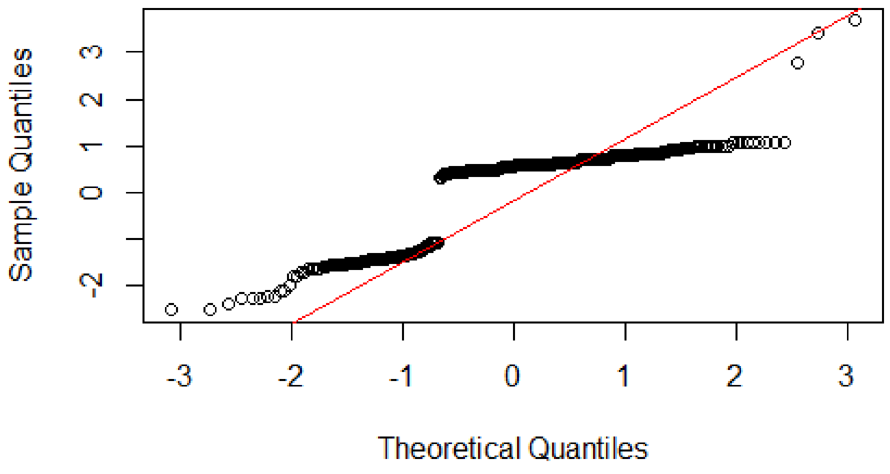

Normal Q-Q Plot of Model IV from Table 3

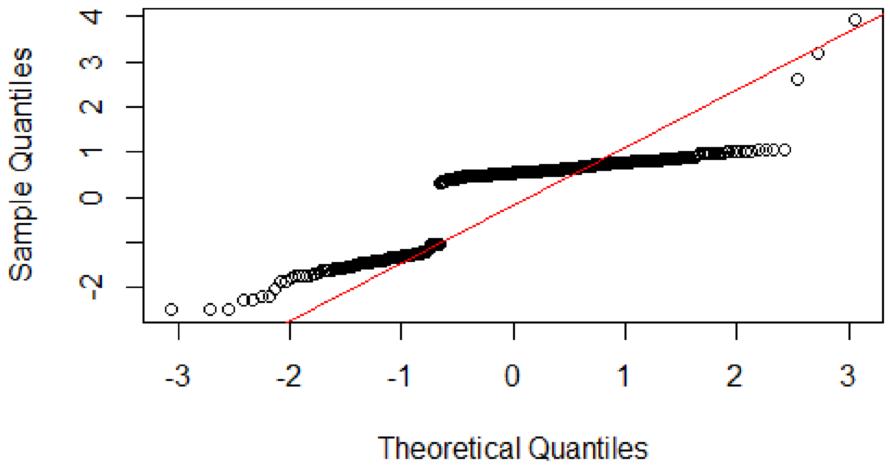

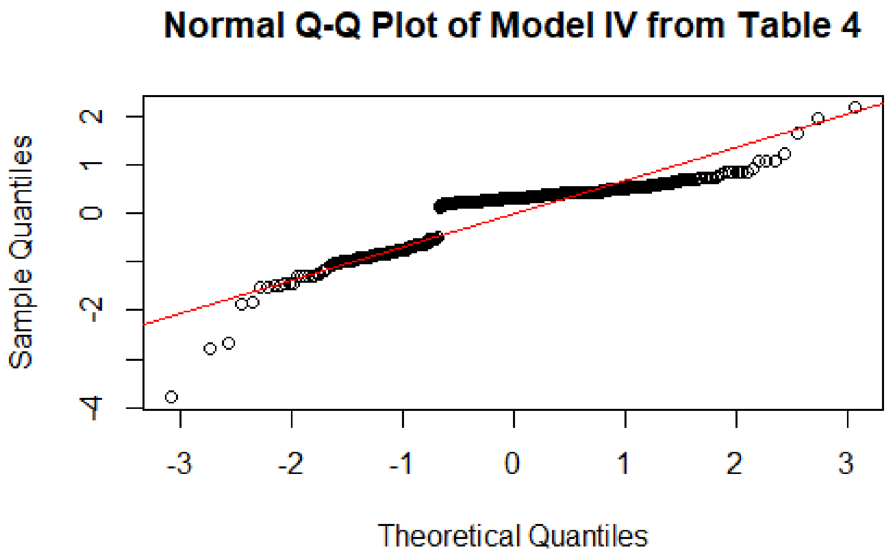

Missingness Plots for Model II when the outcome is not missing

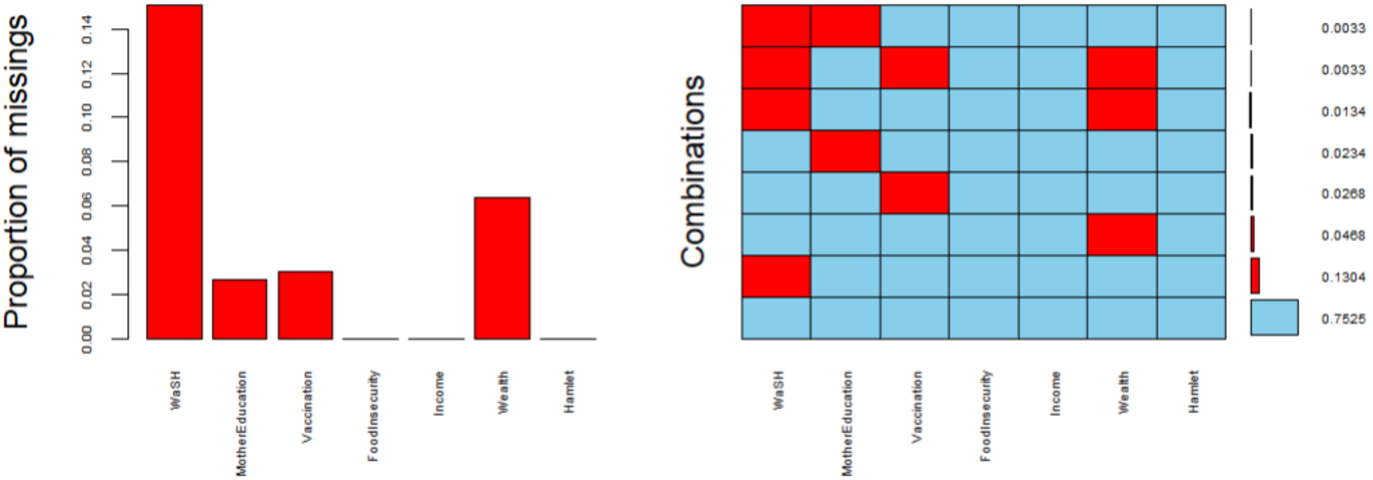

Missingness Plots for Model IV when the outcome is not missing

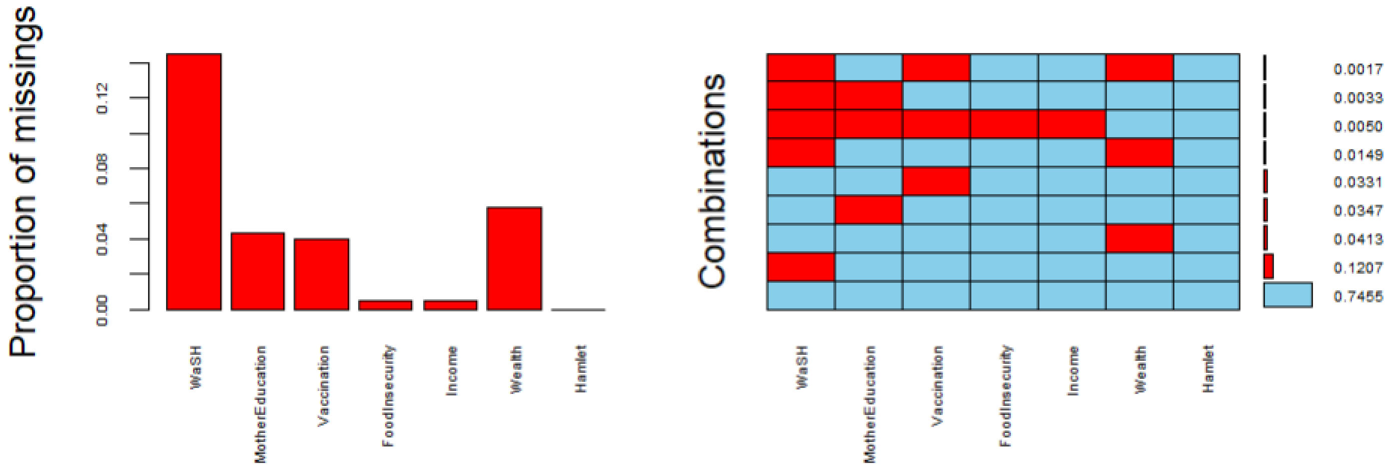

Outcome = b1 * wash + b2 * food + b3 * education

Outcome = food, education
